# Venlafaxine as an Adjuvant Therapy for Inflammatory Bowel Disease Patients With Anxious and Depressive Symptoms: A Randomized Controlled Trial

**DOI:** 10.3389/fpsyt.2022.880058

**Published:** 2022-05-19

**Authors:** Chang Liang, Pingrun Chen, Yu Tang, Chuheng Zhang, Na Lei, Ying Luo, Shihao Duan, Yan Zhang

**Affiliations:** ^1^Department of Gastroenterology, West China Hospital of Sichuan University, Chengdu, China; ^2^Institute for Interdisciplinary Information Science, Tsinghua University, Beijing, China

**Keywords:** antidepressants, quality of life, IBD – inflammatory bowel disease, disease activity, depression, anxiety

## Abstract

**Background and Aims:**

The effect of antidepressant therapy on Inflammatory Bowel Disease (IBD) remains controversial. This trial aimed to assess whether adding venlafaxine to standard therapy for IBD improved the quality of life (QoL), mental health, and disease activity of patients with IBD with anxious and depressive symptoms.

**Methods:**

A prospective, randomized, double-blind, and placebo-controlled clinical trial was conducted. Participants diagnosed with IBD with symptoms of anxiety or depression were randomly assigned to receive either venlafaxine 150 mg daily or equivalent placebo and followed for 6 months. Inflammatory Bowel Disease Questionnaire (IBDQ), Mayo score, Crohn's disease activity index (CDAI), Hospital Anxiety and Depression Scale (HADS), and blood examination were completed before the enrollment, during, and after the follow-up. Mixed linear models and univariate analyses were used to compare groups.

**Results:**

Forty-five patients with IBD were included, of whom 25 were randomized to receive venlafaxine. The mean age was 40.00 (SD = 13.12) years old and 25 (55.6%) were male. Venlafaxine showed a significant improvement on QoL (*p* < 0.001) and disease course (*p* = 0.035), a greater reduction in HADS (anxiety: *p* < 0.001, depression: *p* < 0.001), Mayo scores (*p* < 0.001), and CDAI (*p* = 0.006) after 6 months. Venlafaxine had no effect on IL-10 expression, endoscopic scores, relapse rate, and use rate of biologics and corticosteroids, but did reduce serum level of erythrocyte estimation rate (ESR; *p* = 0.003), C-reactive protein (CRP; *p* < 0.001) and tumor necrosis factor-α (TNF-α; *p* = 0.009).

**Conclusions:**

Venlafaxine has a significantly beneficial effect on QoL, IBD activity, and mental health in patients with IBD with comorbid anxious or depressive symptoms. (Chinese Clinical Trial Registry, ID: ChiCTR1900021496).

## Introduction

Inflammatory bowel diseases (IBD) including Crohn's disease (CD) and ulcerative colitis (UC) are chronic disorders with accelerating incidence associated with psychological disorders, impaired quality of life (QoL), and increased healthcare use (1–3). Numerous studies have shown a strong relationship between IBD and depression and/or anxiety. The prevalence of depression and anxiety in patients with IBD was much higher than that in normal population (4–6), and patients with active IBD showed higher rates of depression and anxiety than those in remission (4, 7, 8). A recent systematic review and meta-analysis (9) revealed that up to a third of patients with IBD were affected by anxiety symptoms and a quarter were affected by depression symptoms. Meanwhile, patients with CD had higher odds of anxiety and depression symptoms than patients with UC, and women with IBD were more likely to have symptoms of anxiety than men. Psychological factors, such as depression and anxiety, may exert a negative influence on the course of IBD, including a higher rate of relapse, surgery, and hospitalization, and higher use rate of steroids or biologics (8, 10–13). A recent meta-analysis (14) reported that anxiety was associated with significantly higher risks of escalation of therapy, hospitalization, and emergency department attendance. Depression was associated with higher risks of flare, escalation of therapy, hospitalization, emergency department attendance, and surgery. Recent research revealed that patients with a history of depression were more likely to be diagnosed with IBD, but can be protected by antidepressant treatments (15). Antidepressants were reported to ameliorate disease severity in patients with IBD with psychiatric disorders (16–20). Furthermore, it is observed that the anti-inflammatory properties of antidepressants may directly influence the inflammatory response (21, 22). However, most of the studies were retrospective and observational in design, and the randomized clinical trials (RCTs) are limited. A pilot RCT of fluoxetine in 26 patients with CD did not show any advantage in terms of maintenance of disease remission or psychological wellness (23). Till now, the current outcomes are inconsistent, and no clear conclusions can be formed on the efficacy and safety of antidepressants in IBD.

Some clinicians have indicated that use of serotonin and norepinephrine reuptake inhibitors (SNRIs) may play a more effective role on immunoregulation than selective serotonin reuptake inhibitors (SSRIs) in patients (24). Moreover, venlafaxine has better effects for depression than other five first-line antidepressants (fluoxetine, paroxetine, escitalopram, sertraline, and fluvoxamine) (25). Some research indicated that venlafaxine has both immunoregulatory activity and anti-inflammation effects (26–30). At present, there is no report on the clinical treatment of venlafaxine on patients with IBD with anxious or depressive symptoms. Therefore, we conducted a prospective RCT to evaluate whether the adjunctive therapy with venlafaxine can bring more benefit to patients with IBD with anxious and/or depressive symptoms than placebo under routine IBD treatment as usual.

## Methods

### Study Design and Patients

This was a prospective, double-blind placebo RCT to evaluate the efficacy of venlafaxine added to standard therapy (31) for patients with IBD with symptoms of anxiety and depression as compared to placebo. Patients were recruited from the outpatient clinic and inpatient of the Department of Gastroenterology and Hepatology at the West China Hospital of Sichuan University, the largest IBD center in southwest China, between April 2019 and May 2020. The diagnosis of CD and UC was based on the third European Evidence-based Consensus on Diagnosis and Management of Crohn's disease and Ulcerative Colitis (32, 33), and disease extent was defined according to the Montreal classification (34). Psychiatric diagnoses was undertaken using the Structural Clinical Interview for DSM disorders (SCID) (35). The study was approved by the Biomedical ethics committee of West China Hospital, Sichuan University and carried out in accordance with the Helsinki Declaration. The trial was registered at Chinese Clinical Trial Registry: trial identifier ChiCTR1900021496. All authors had access to the study data and reviewed and approved the final manuscript.

The inclusion criteria were (1) age 18–65 years, (2) diagnosis of UC or CD based on the combination of clinical, endoscopic, and histologic investigations, (3) patients with IBD with mild, moderate, or severe disease severity [defined by CDAI and Mayo score (36)], (4) consistent use of the same medications for IBD, including mesalazine corticosteroids (<15 mg), immunomodulators, and biologics (>4 weeks), (5) with a Hospital Anxiety and Depression Scale (HADS) score ≥ 8 on one or both subscales, and (6) willingness to participate in the intervention and complete the study.

The exclusion criteria were (1) patients with serious uncontrolled mental illness, alcohol or substance-dependence, and cognitive impairment, (2) patients who are taking or have taken antidepressants or psychotherapy within 6 months, (3) with other diseases that are highly associated with depression or anxiety, such as cardiovascular disease, cancer, and multiple sclerosis, (4) pregnant, breastfeeding, or preparing for pregnancy, (5) patients taking any medications listed as contraindicated with venlafaxine, (6) with an ileostomy or colostomy, (7) change in IBD medication, including use of steroids (prednisolone > 15 mg or equivalent) within 3 months, and (8) patients with severe disease (e.g., recent major surgery, hepatic and renal dysfunction and/or complicated disease).

### Intervention and Randomization

Patients were randomized to receive either venlafaxine at a fixed time of day or equivalent placebo which was a gelatin capsule filled with starch. To improve the participant tolerance to venlafaxine, the dosage was directed by a professional psychiatrist. That is, 75 mg venlafaxine (sustained-release form) was daily taken for the first week, then was changed into 150 mg till the end of the experiment before it was restored to its initial dosage level (37). Patients in each group remained on their current IBD medication. The intervention lasted for 6 months. The randomization was conducted by using computer-generated random numbers. The randomization process was anonymized and performed by the second author who packed random allocation sequences into opaque envelopes. The therapists and participants were blinded to the intervention. Participants were told that the aim of the trial was to determine if venlafaxine is an effective complementary therapy for IBD and that they would be assigned to one of the two groups: venlafaxine group and placebo group. All participating patients signed informed consent form to participate.

### Study Endpoints

Participants were required to provide blood samples and complete corresponding investigations and questionnaires on three occasions (baseline, and at 3 and 6 months). At baseline, participants were asked to report their demographics and clinical status. These data included age, age at diagnosis, gender, marital status, tobacco use, disease phenotype, disease activity scores [Crohn's disease activity index (CDAI) (38, 39) for CD and Mayo score for UC (40)], disease course, IBD-related surgery, endoscopy evaluation [Simple Endoscopic Score for Crohn's disease (SES-CD) (41) and Ulcerative Colitis Endoscopic Index of Severity (UCEIS) (42)], medication, body mass index (BMI), and laboratory data. Blood samples were taken for the measurement of the level of white blood count (WBC, reference value: 3.5–9.5 × 10^9^/L), thrombocyte (reference value: 100–300 × 10^9^/L), hemoglobin (reference value: males 130–175 g/L; females 115–150 g/L), aspartate aminotransferase (reference value: <35 IU/L), alanine aminotransferase (reference value: <40 IU/L), albumin (ALB, reference value: 40.0–55.0 g/L), creatinine (reference value: 48–79 μmol/L), erythrocyte sedimentation rate (ESR, reference value: <21 mm/h), C-reactive protein (CRP, reference value: <5 mg/L), thyroid-stimulating hormone (TSH, reference value: 0.27–4.2 mU/L), tumor necrosis factor α (TNF-α, reference value: <8.1 pg/ml), and interleukin 10 (IL-10, reference value: 0.0–9.1 pg/ml).

The primary outcome measures of this study were the Inflammatory Bowel Disease Questionnaire (IBDQ) (43) score and disease activity scores (CDAI for CD and Mayo score for UC) at the outset, 3 months, and 6 months, which were analyzed in the complete case population.

The secondary outcome measures were the means on the Hospital Anxiety Depression Scale (HADS) (44), disease course, SES-CD, UCEIS, relapse rate, frequency of corticosteroids/biologics use, and laboratory parameters (WBC, ALB, CRP, ESR, TNF-α, IL-10) between the venlafaxine and placebo groups. The HADS was measured on three occasions while the rest secondary outcomes were measured at the outset and 6 months.

Release was defined as a worsening of bowel function and rectal bleeding with an endoscopic grade of 2, 3, or 4 for UC (45). A score of > 150 combined with a rise of 100 points has been considered indicative of a relapse for CD (46).

The IBDQ is a validated QoL assessment tool specifically for patients with IBD (47). It contains 32 questions with the range of 32 to 224 and has a minimum clinically significant change score of 20 points (48). The questions are split into 4 domains including bowel symptoms, systemic symptoms, social functioning, and emotional functioning, and a mean score can be calculated for each domain where higher scores indicate a better QoL.

The HADS was found to perform well in assessing the symptom severity and caseness of anxiety disorders and depression. Scores of 8 or higher are considered to have anxious or depressive state (49).

### Sample Size

Sample size calculations were based on three primary outcome measures of CDAI, Mayo Score, and IBDQ using the PASS 15 software package. A power analysis was performed using α = 0.05 and β = 0.80. To assume an expected decrease of 70 points of CDAI (50), a drop of 3 points of Mayo score (51) (which are defined the minimum clinical responses), and an increase of 20 points of IBDQ (48) at 6 months in the venlafaxine group vs. no effect in the placebo group, the maximal required sample size was determined to be 22 patients in each group. A dropout rate of approximately 10% was predicted. Hence, 50 patients were scheduled to recruit in the study.

### Statistical Analysis

SPSS 25.0 and GraphPad Prism 7.0 software were used to perform the statistical analyses. The independent samples *t*-test and the chi-square tests were used for baseline demographic disease variables. Continuous variables were presented as mean values (standard deviation, SD), while categorical variables were presented as percentages and absolute numbers.

The linear mixed model was used to assess differences in main outcome measures in venlafaxine group vs. control group. The venlafaxine and placebo group were compared in the total IBD group. Then, separate analyses were performed in the UC and CD group. At first, saturated models were set with IBDQ, disease activity, HADS-Anxiety, HADS-Depression, Mayo score, CDAI, UCEIS, SES-CD, WBC, ALB, CRP, ESR, TNF-α, and IL-10 as dependent variables. The fixed effects included age, gender, intervention, medication, time, and time interaction with treatment. Deviance statistic using Akaike information criterion (AIC) (52) was applied to determine the covariance structure. Then, the final models were by eliminating the insignificant fixed effects. The significance of the difference between the saturated models and the final models were determined with AIC. The results were considered significant results with the two-sided *p* value of <0.05. Effect sizes were calculated by dividing the effects by the estimated SDs at baseline. All authors had access to the study data and reviewed and approved the final manuscript.

## Results

### Participant Flow Diagram

**Figure 1:**
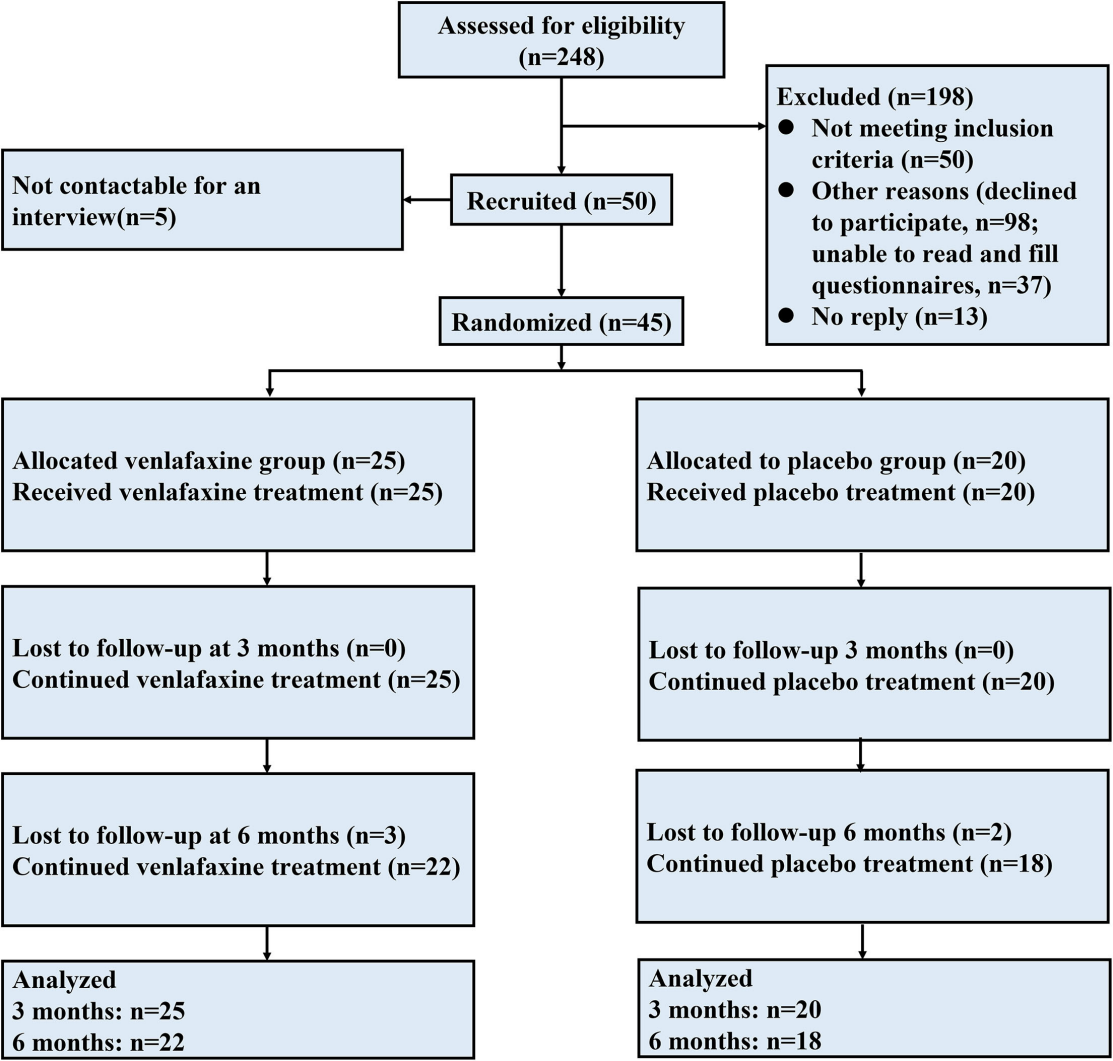
Flowchart of the intervention.

Figure 1 illustrates an overview of participant flow. Overall, 45 patients were randomized in the study: 25 in the venlafaxine group and 20 in the placebo group. Twenty-two patients were diagnosed with UC [11 (50%) in venlafaxine group], and 25 patients were diagnosed with CD [14 (56%) in the venlafaxine group]. After a 3-month following, three patients in venlafaxine group discontinued intervention due to adverse events (AEs, 2 for dizziness and 1 for insomnia) and two patients in placebo were no longer to participate after 3 months. Endpoints were defined as follows: patients were followed up for 6 months, lost to follow-up, or withdrew from trial after inclusion.

### Baseline Characteristics

Supplementary Table 1 presents demography and clinical and treatment characteristics in each group. Baseline characteristics were similar between the two groups with no statistically significant differences in age, gender, marital status, tobacco use, disease activity, Montreal classification, surgery rate, medication, and BMI in both the entire IBD population and its subtypes (all *p* > 0.05). Fourteen patients with CD and 11 patients with UC were randomized to the venlafaxine group, whereas 9 patients with CD and 11 patients with UC were randomized to the control group (Fisher's exact test *p* = 0.463). The mean age was 40.00 (SD = 13.12) years and 20 (44.4%) were female. The laboratory parameters including WBC, ALB, CRP, ESR, TNF-a, and IL-10 showed no differences between group (all *p* > 0.05). Mayo scores and CDAI score were similar in the venlafaxine and placebo groups (Mayo: 6.64 vs. 5.27, *p* = 0.412, CDAI: 261.43 vs. 171.78, *p* = 0.847). There was no difference in the medications for IBD between the venlafaxine group and placebo group (*p* = 0.891). Moreover, no significant differences were found in HADS and IBDQ scores between venlafaxine and placebo groups at baseline (Supplementary Table 2).

### Outcomes

Table 1 reveals the corrected outcomes after the line mixed model analysis in entire IBD and subgroups. Tables 2–4 present estimates and effects size in IBD, UC, and CD, respectively. Table 5 shows the outcomes of disease course, relapse rate, endoscopic scores, and use rate of steroids or biologics by univariate analyses.

**Table 1:** The final mixed linear model in inflammatory bowel disease (IBD) population, ulcerative colitis (UC), and Crohn's disease (CD) subgroups.

**Outcome effect**	**IBD**			**UC**			**CD**		
	**Estimate**	**SE**	***p***	**Estimate**	**SE**	***p***	**Estimate**	**SE**	***p***
IBDQ									
Intercept	168.86	14.85	<0.001	143.63	7.91	<0.001	175.29	14.05	<0.001
Time	−0.99	2.28	0.663	−0.75	2.60	0.775	3.42	2.38	0.152
Treatment × time	17.85	2.96	<0.001	22.72	3.55	<0.001	9.46	3.01	0.002
Age	−0.63	0.36	0.074	–	–	–	−0.86	0.35	0.014
Mayo score									
Intercept	–	–	–	5.80	0.70	<0.001	–	–	–
Treatment × time	–	–	–	−1.60	0.35	<0.001	–	–	–
Time	–	–	–	−0.11	0.30	0.716	–	–	–
CDAI									
Intercept	–	–	–	–	–	–	268.02	24.03	<0.001
Treatment × time	–	–	–	–	–	–	−44.23	13.51	0.001
Time	–	–	–	–	–	–	−16.78	11.50	−1.459
HADS Anxiety									
Intercept	10.09	0.26	<0.001	9.81	0.46	<0.001	10.55	0.50	<0.001
Time	−0.06	0.18	0.740	−1.26	0.31	<0.001	−1.26	0.41	0.002
Treatment × time	−2.82	0.22	<0.001	−1.26	0.34	<0.001	−1.43	0.48	0.003
HADS Depression									
Intercept	7.07	0.92	<0.001	8.83	0.71	<0.001	5.54	1.30	<0.001
Treatment	–	–	–	−1.03	1.01	0.306	–	–	–
Time	0.00	0.22	0.998	−1.23	0.28	<0.001	−0.34	0.42	0.422
Treatment × time	−2.40	0.26	<0.001	−0.09	0.39	0.817	−1.58	0.38	<0.001
Age	0.04	0.02	0.042	–	–	–	0.07	0.03	0.009
CRP									
Intercept	21.48	2.47	<0.001	8.90	2.96	0.003	15.59	2.57	<0.001
Treatment	–	–	–	7.81	4.19	0.062	–	–	–
Time	1.04	0.96	0.276	−2.34	1.52	0.123	−0.43	1.48	0.771
Treatment × time	−6.21	1.16	<0.001	−2.48	2.14	0.246	−4.35	1.59	0.006
Biologics × time	−7.05	3.95	0.075	–	–	–	–	–	–
ESR									
Intercept	26.65	5.18	<0.001	17.55	4.80	<0.001	40.64	5.38	<0.001
Treatment	10.97	6.95	0.114	10.22	6.25	0.102	–	–	–
Time	1.54	1.91	0.422	−2.23	2.05	0.278	1.09	3.04	0.721
Treatment × time	−12.21	2.57	<0.001	−3.36	2.97	0.257	−13.55	3.30	<0.001
Biologics × time	–	–	–	−0.91	3.42	0.790	–	–	–
TNF-α									
Intercept	45.49	26.38	0.085	6.05	13.71	0.659	69.24	30.99	0.025
Treatment	–	–	–	47.95	19.38	0.013	–	–	–
Time	−1.93	4.67	0.679	0.18	2.12	0.932	−7.91	5.73	0.167
Treatment × time	−12.53	5.91	0.034	−7.26	3.16	0.022	−10.49	5.57	0.059
Biologics × time	–	–	–	−38.19	5.54	<0.001	–	–	–
IL−10									
Intercept	3.01	1.56	0.053	4.38	1.88	0.020	3.47	0.33	<0.001
Time	−0.07	0.58	0.900	1.50	0.62	0.015	0.16	0.15	0.304
Treatment × time	0.32	0.74	0.661	−0.69	0.64	0.278	0.11	0.19	0.304
Gender	4.65	2.24	0.038	2.90	1.82	0.112	–	–	–

*IBD, inflammatory bowel disease; UC, ulcerative colitis; CD, Crohn's disease; SE, standard error; IBDQ, inflammatory bowel disease questionnaire; CDAI, Crohn's Disease Activity Index; HADS, Hospital Anxiety and Depression Scale; C-reactive protein; ESR, erythrocyte sedimentation rate; TNF-a, tumor necrosis factor α; IL-10, interleukin 10*.

**Table 2:** Estimates and effects sizes for venlafaxine group and placebo group in IBD population.

**Outcome**	**IBD Placebo**		**IBD Venlafaxine**		**Ven vs Con**	***p***
	**Estimate**	**Effect size**	**Estimate**	**Effect size**	**Effect size**	
IBDQ						
Baseline	143.52		143.52			
3 months	142.53	−0.03	160.38	0.55	0.59	0.005
6 months	141.53	−0.04	177.24	0.50	1.19	<0.001
HADS Anxiety						
Baseline	10.09		10.09			
3 months	10.03	−0.04	7.22	−1.52	−1.52	<0.001
6 months	9.97	−0.03	4.34	−1.36	−1.36	<0.001
HADS Depression						
Baseline	8.81		8.81			
3 months	8.81	0.00	6.41	−1.47	−1.44	<0.001
6 months	8.81	0.00	4.00	−1.23	−2.27	<0.001
CRP						
Baseline	17.34		17.34			
3 months	18.23	0.06	12.01	−0.58	−0.53	0.018
6 months	19.12	0.05	6.69	−0.89	−1.24	<0.001
ESR						
Baseline	26.65		37.62			
3 months	28.18	0.07	26.95	−0.55	−0.06	0.779
6 months	29.72	0.06	16.27	−0.75	−0.69	0.003
TNF-a						
Baseline	49.95		49.95			
3 months	47.85	−0.04	35.62	−0.23	−0.22	0.304
6 months	45.76	−0.04	21.29	−0.34	−0.55	0.009
IL−10						
Baseline	5.07		5.07			
3 months	5.33	0.02	5.64	0.30	0.06	0.894
6 months	5.58	0.01	6.21	0.36	0.12	0.825

*IBD, inflammatory bowel disease; Ven, venlafaxine; Con, control; IBDQ, inflammatory bowel disease questionnaire; HADS, Hospital Anxiety and Depression Scale; CRP, C-reactive protein; ESR, erythrocyte sedimentation rate; TNF-a, tumor necrosis factor α; IL−10, interleukin 10*.

**Table 3:** Estimates and effects sizes for venlafaxine group and placebo group in UC.

**Outcome**	**UC Placebo**		**UC Venlafaxine**		**Ven vs Con**	***p***
	**Estimate**	**Effect size**	**Estimate**	**Effect size**	**Effect size**	
IBDQ						
Baseline	143.63		143.63			
3 months	142.88	−0.02	165.60	0.55	0.61	0.055
6 months	142.14	−0.02	187.57	0.52	1.27	<0.001
Mayo score						
Baseline	5.80		5.80			
3 months	5.69	−0.03	4.09	−0.88	−0.64	0.054
6 months	5.59	−0.03	2.38	−1.13	−1.47	<0.001
HADS Anxiety						
Baseline	9.81		9.81		−1.07	0.005
3 months	8.55	−1.79	7.30	−1.28	−1.40	<0.001
6 months	7.30	−0.71	4.78	−1.39		
HADS Depression						
Baseline	8.83		7.80			
3 months	7.61	−0.74	6.48	−0.89	−0.71	0.018
6 months	6.38	−0.82	5.17	−0.89	−0.81	0.007
CRP						
Baseline	8.90		16.71			
3 months	6.56	−0.81	11.89	−0.50	1.01	0.012
6 months	4.22	−0.57	7.07	−0.94	0.62	0.043
ESR						
Baseline	15.73		26.09			
3 months	13.34	−0.28	20.50	−0.32	0.59	0.086
6 months	10.94	−0.37	14.92	−0.65	0.53	0.083
TNF-a						
Baseline	6.05		54.00			
3 months	2.75	−1.16	43.46	−0.20	3.35	<0.001
6 months	−0.55	−0.98	32.91	−0.24	2.74	<0.001
IL−10						
Baseline	5.82		5.82			
3 months	7.36	0.07	6.68	0.47	−0.11	0.881
6 months	8.89	0.06	7.54	0.55	−0.21	0.804

*UC, ulcerative colitis; Ven, venlafaxine; Con, control; IBDQ, inflammatory bowel disease questionnaire; HADS, Hospital Anxiety and Depression Scale; CRP, C-reactive protein; ESR, erythrocyte sedimentation rate; TNF-a, tumor necrosis factor α; IL−10, interleukin 10*.

**Table 4:** Estimates and effects sizes for venlafaxine group and placebo group in CD.

**Outcome**	**CD Placebo**		**CD Venlafaxine**		**Ven vs Con**	***p***
	**Estimate**	**Effect size**	**Estimate**	**Effect size**	**Effect size**	
IBDQ						
Baseline	142.39		142.39			
3 months	145.81	0.15	155.27	0.60	0.43	0.150
6 months	149.22	0.14	168.14	0.57	0.81	0.006
CDAI						
Baseline	268.02		268.02			
3 months	251.24	−0.16	207.00	−0.66	−0.44	0.134
6 months	234.46	−0.13	145.99	−0.77	−0.87	0.006
HADS Anxiety						
Baseline	10.55		10.55			
3 months	9.29	−0.55	7.86	−1.48	−0.70	0.019
6 months	8.03	−0.60	5.17	−1.00	−1.20	<0.001
HADS Depression						
Baseline	8.38		8.38			
3 months	8.04	−0.16	6.45	−1.17	−0.85	0.005
6 months	7.70	−0.18	4.53	−0.71	−1.42	<0.001
CRP						
Baseline	15.59		15.59			
3 months	15.16	−0.05	10.81	−0.53	−0.48	0.102
6 months	14.73	−0.03	6.04	−0.71	−0.95	0.003
ESR						
Baseline	40.64		40.64			
3 months	41.72	0.06	28.18	−0.64	−0.74	0.013
6 months	42.81	0.05	15.72	−0.70	−1.33	<0.001
TNF-a						
Baseline	63.58		63.58			
3 months	55.58	−0.14	45.24	−0.26	−0.16	0.584
6 months	47.57	−0.15	26.89	−0.45	−0.44	0.142
IL−10						
Baseline	3.47		3.47			
3 months	3.63	0.11	3.74	0.14	0.07	0.824
6 months	3.78	0.14	4.01	0.16	0.16	0.592

*CD, Crohn's disease; Ven, venlafaxine; Con, control; IBDQ, inflammatory bowel disease questionnaire; CDAI, Crohn's Disease Activity Index; HADS, Hospital Anxiety and Depression Scale; CRP, C-reactive protein; ESR, erythrocyte sedimentation rate; TNF-a, tumor necrosis factor α; IL−10, interleukin 10*.

**Table 5:** Disease course, relapse rate, endoscopic scores, and use rate of biologics and corticosteroids in patients with IBD, randomized to venlafaxine group or placebo group assessed at baseline and 6 months.

	**Venlafaxine**		**Placebo**		***p***
	**Baseline 6 months**		**Baseline 6 months**		
Disease course, *n* (%)					
Remission	5 (20.0)	8 (36.4)	3 (15.0)	3 (16.7)	0.035
Mild	3 (12.0)	12 (54.5)	8 (40.0)	9 (50.0)	
Moderate	16 (64.0)	2 (9.1)	8 (40.0)	5 (27.8)	
Severe	1 (4.0)	0 (0.0)	1 (5.0)	1 (5.5)	
Relapse, *n* (%)	3 (12.0)	1 (4.5)	3 (15.0)	5 (27.8)	0.074
UCEIS, mean (SD)	3.7 (3.2)	2.7 (1.9)	3.9 (2.6)	3.7 (1.7)	0.249
SES-CD, mean (SD)	7.2 (4.0)	4.6 (4.1)	11.9 (4.5)	8.6 (4.2)	0.071
Biologics, *n* (%)	10 (40.0)	11 (50.0)	6 (30.0)	9 (50.0)	>0.999
Corticosteroids, *n* (%)	1 (4.0)	1 (4.5)	2 (10.0)	4 (22.2)	0.250

*CDAI, Crohn's Disease Activity Index; UCEIS, Ulcerative Colitis Endoscopic Index of Severity; SES-CD, Simple Endoscopic Score for Crohn's disease; WBC, white blood count; ALB, albumin; CRP, C-reactive protein; ESR, erythrocyte sedimentation rate; TNF-a, tumor necrosis factor α; IL−10, interleukin 10; HADS, Hospital Anxiety and Depression Scale; IBDQ, inflammatory bowel disease questionnaire*.

#### Primary Outcomes

##### IBDQ Scores

**Figure 2:**
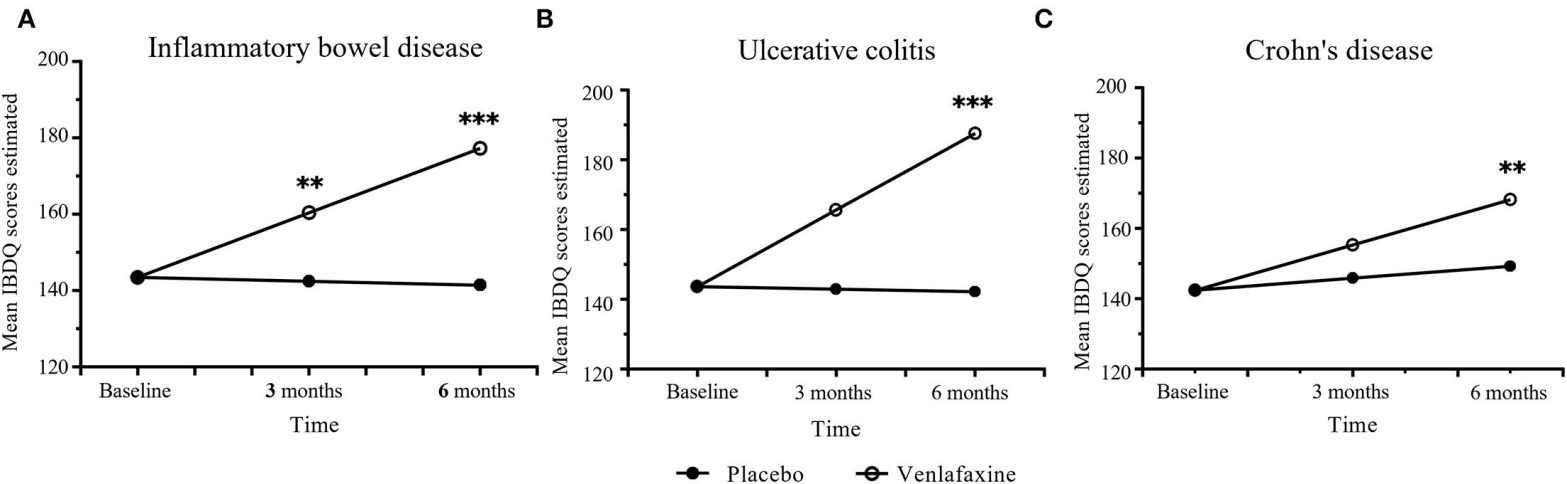
Primary outcomes of mean Inflammatory Bowel Disease Questionnaire (IBDQ) scores between venlafaxine group and placebo group. IBDQ analyzed in entire IBD group **(A)**, ulcerative colitis (UC) group **(B)**, and Crohn's disease (CD) group **(C)**. Effect size between the venlafaxine group and placebo group is significant with *p < 0.05, **p < 0.01 and ***p < 0.001.

Regarding the improvement in IBDQ scores, the mixed linear model showed significant interaction between treatment and time in the IBD population and UC and CD groups, while age effect is only significant in CD group (Table 1). The effect for IBDQ was medium at 3 months and increased at 6 months (Table 2). IBDQ scores were significantly higher in the venlafaxine group compared with placebo group in IBD group (3 months: comparison of effect size:0.59, *p* = 0.005; 6 months: comparison of effect size: 1.19, *p* <0.001; Figure 2).

##### Mayo Score and CDAI Scores

**Figure 3:**
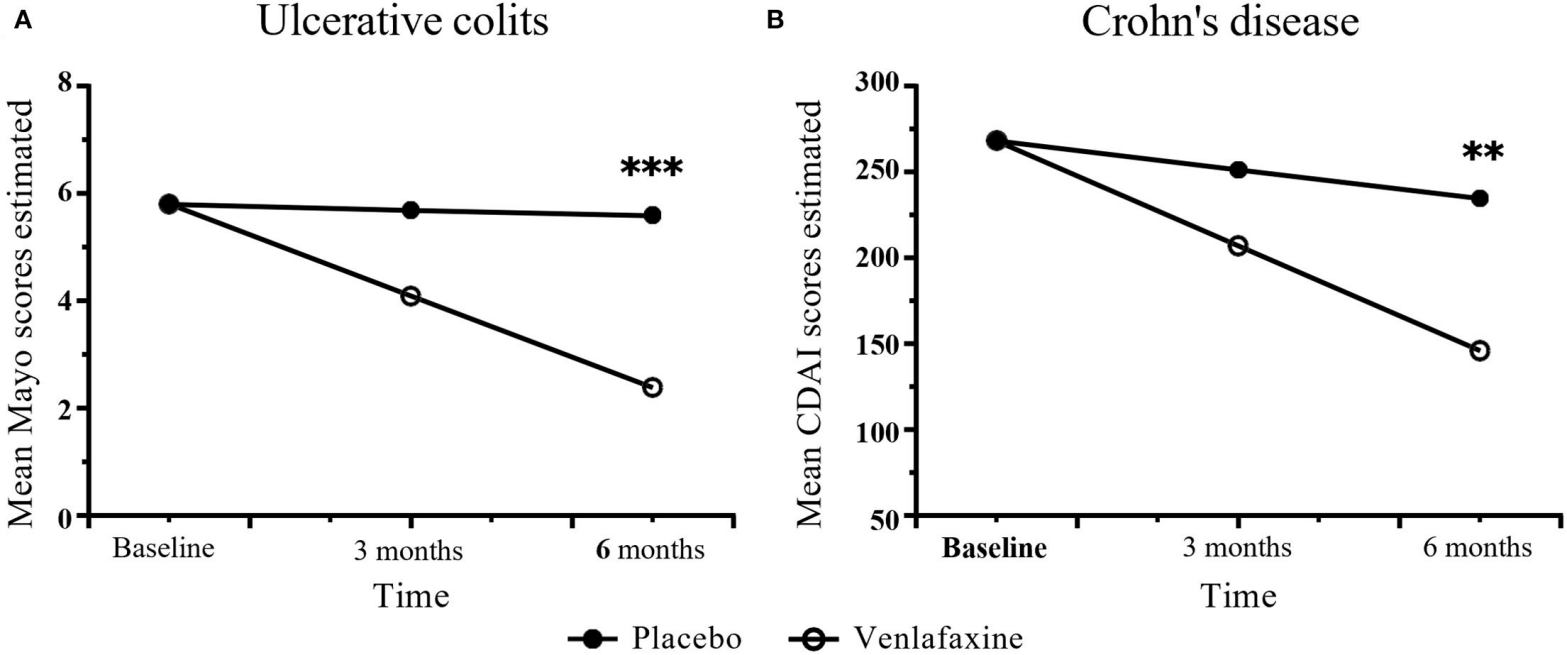
Disease activity measured with Mayo score in UC **(A)** and Crohn's Disease Activity Index (CDAI) score in CD **(B)** during the study period in venlafaxine group and placebo group. Effect size between the venlafaxine group and placebo group is significant with **p < 0.01 and ***p < 0.001.

For disease activity index, UC was analyzed by Mayo score, while CD by CDAI. There was no difference in Mayo scores (comparison of effect size: −0.64, *p* = 0.055) between venlafaxine group and placebo group at 3 months. However, patients with UC with venlafaxine had lower Mayo score than that in placebo group after the 6-month assessment (comparison of effect size: −1.47, *p* <0.001) (Table 3; Figure 3A). For the CD group, no significant difference was observed in the CDAI score in venlafaxine group vs. the control group at 3 months (comparison of effect size: −0.44, *p* = 0.134), but venlafaxine showed significant decrease in CDAI scores compared with placebo at 6 months (comparison of effect size: −0.87, *p* = 0.006) (Table 4; Figure 3B).

#### Secondary Outcomes

##### Hospital Anxiety and Depression Scale

It was clear that the HADS-anxiety model had a significant treatment x time effect in IBD population and significant treatment x time effect and time effect in subgroups. The HADS-depression model showed significant treatment x time and age effect in the IBD and CD groups (Table 1). In IBD populations, statistical significance was found in HADS anxiety scores between the venlafaxine group and control group at 3 months and at 6 months, respectively (estimated at 3 months: 7.22 vs. 10.03, *p* <0.001; estimated at 6 months: 4.34 vs. 9.97, *p* <0.001). Similarly, a significant reduction in the HADS depression scores was observed between two groups both at 3 months (estimated at 3 months: 6.41 vs. 8.81, *p* <0.001) and at 6 months (estimated at 6 months: 4.00 vs. 8.81, *p* <0.001) (Table 2).

##### Laboratory Parameters

In IBD population, serum level of CRP, ESR, and TNF-a were decreased in the venlafaxine group during the period of 6 months. There was a significant difference in CRP level between venlafaxine group and placebo group at 3 months (estimate: 12.01 vs. 18.23, *p* = 0.018) and 6 months (estimate: 6.69 vs. 19.12, *p* <0.001). A significant reduction of the level of ESR (estimate: 16.27 vs. 29.72, *p* = 0.003) and TNF-α (estimate: 21.29 vs. 45.76, *p* = 0.009) was presented at 6 months. For IL-10, there were no differences between two groups (Table 2).

##### Disease Course and Relapse Rate

The intervention improved disease severity (Table 5). There were statistical differences in the ratios of disease course at 6 months (*p* = 0.035). We also compared the disease severity between the four groups taking venlafaxine (*p* = 0.021), indicating that the patients in active phases of IBD responded better to venlafaxine than those in remission. However, there was no statistically significant difference in the relapse rate between two groups at 6 months in IBD population (4.5 vs. 27.8%, *p* = 0.074).

##### Endoscopic Scores

Ulcerative Colitis Endoscopic Index of Severity (1.9 vs. 1.7, *p* = 0.249) and SES-CD (4.6 vs. 8.6, *p* = 0.071) showed no significant differences between the venlafaxine group and placebo group at 6 months (Table 5).

##### Medication Use

The frequency of corticosteroid use was decreased in the venlafaxine group and was increased in the control group with no significant difference between the two groups after 6-month therapy (venlafaxine: 4.0–0.0%, control: 10.0–22.2%, *p* = 0.250). The frequency of biologics use was similar between venlafaxine and placebo group (venlafaxine: 40.0–50.0%, control: 30.0–50.0%, *p*>0.999) (Table 5).

#### Subtype Analysis

##### IBDQ Scores

When analyzed in subtypes, no significant differences were found between the venlafaxine and control group in UC and CD, respectively, at 3 months (UC: comparison of effect size: 0.61, *p* = 0.055; CD: comparison of effect size:0.43, *p* = 0.150). However, at 6 months, patients with UC prescribed with venlafaxine showed greater improvement in IBDQ compared with patients with UC in the placebo group (comparison of effect size: 1.27, *p* <0.001). Also, patients with CD who were taking venlafaxine had higher IBDQ than that in placebo group (comparison of effect size: 0.81, *p* = 0.006) (Tables 3, 4).

##### Hospital Anxiety and Depression Scale

Patients with UC taking venlafaxine tended to have lower HADS-anxiety scores (estimated at 3 months: 7.30 vs. 8.55, *p* = 0.005; estimated at 6 months: 4.78 vs. 7.30, *p* <0.001) and HADS-depression scores (estimated at 3 months: 6.48 vs. 7.61, *p* = 0.018; estimated at 6 months: 5.17 vs. 6.38, *p* = 0.007) (Table 3). Patients with CD in the venlafaxine group also had lower HADS-anxiety scores (estimated at 3 months: 7.86 vs. 9.29, *p* = 0.019; at 6 months: 5.17 vs. 8.03, *p* <0.001) and HADS-depression scores (estimated at 3 months: 6.45 vs. 8.04, *p* = 0.005; estimated at 6 months: 4.53 vs. 7.70, *p* <0.001) (Table 4).

##### Laboratory Parameters

In UC, the TNF-α model showed the significant treatment effect (*p* = 0.013), treatment x time effect (*p* = 0.022), and biologics x time effect (*p* <0.001), which meant that venlafaxine and biologics can both affect the level of TNF-α (Table 1). Venlafaxine significantly reduced TNF-α serum level compared with placebo group both at 3 (estimate: 43.46 vs. 2.75, *p* <0.001) and 6 months (estimate: 32.91 vs.0.55, *p* <0.001). In addition, patients with UC in the venlafaxine group tended to have a significant reduction in CRP level compared with that in placebo group (estimated at 3 months: 11.89 vs. 6.56, *p* = 0.012; estimated at 6 months: 7.07 vs. 4.22, *p* = 0.043) (Table 3). Patients with CD in the venlafaxine group had lower serum level of CRP compared with placebo group (CRP: estimated at 3 months: 10.81 vs. 15.16, *p* = 0.102; at 6 months: 6.04 vs. 14.73, *p* = 0.003) and lower serum level of ESR (estimated at 3 months: 28.18 vs. 41.72, *p* = 0.013; estimated at 6 months: 15.72 vs. 42.81, *p* <0.001) (Table 4).

#### Safety

Overall, there were 7 (28%) participants in the venlafaxine group vs. 2 (10%) in controls reported side-effects, of which two patients withdrew from venlafaxine due to AEs. The remaining patients all resolved during the first 3 weeks of intervention. In the venlafaxine group, side-effects included dizziness (*n* = 3), palpitation (*n* = 1), nausea (*n* = 2), and insomnia (*n* = 1). In the placebo group, side-effects were nausea (*n* = 1) and diarrhea (*n* = 1).

## Discussion

This study is the first longitudinal double-blinded placebo randomized trial to evaluate the efficacy of venlafaxine on QoL, mental health, IBD disease activity, endoscopic scores, relapse rate, frequency of biologics, and corticosteroid use in IBD patients as the adjuvant therapy. The results showed that venlafaxine is effective to improve QoL, alleviated depressive and anxious symptoms, improve CDAI and Mayo score, and reduced the blood level of CRP, ESR, and TNF-α.

A recent review assessed the efficacy and safety of antidepressants for anxiety and depression treatment, the effects of antidepressants on QoL, and disease activity in IBD, but no firm conclusions can be drawn (53). At present, only two studies were double-blind RCTs. One RCT of duloxetine, involving 44 participants, found that patients with IBD who were taking duloxetine had significantly lower depression, anxiety, symptom scores, and significantly greater QoL scores compared with placebo (54). The study was restricted by the short follow-up period (12 weeks) and the small sample size. Another pilot RCT of fluoxetine in 26 patients with CD was also conducted, but it found that fluoxetine had no effect on maintenance of disease remission or psychological well-being (23). This trial was underpowered to detect any difference due to the limited sample size. Patients with CD in remission, of whom previous use of antidepressants and current CD treatment were not controlled, were recruited. Moreover, trials should establish disease activity by multiple measures or at least 2 objective measures. Our study enrolled patients with IBD at various disease severities (classified as mild, moderate, severe, or in remission). We assessed IBD activity by CDAI, Mayo scores, endoscopic scores, relapse rate, serum ESR, CRP, and TNF-α levels. Further, it was argued that if antidepressants could alleviate IBD disease activity via improving the mood based on the current brain–gut–microbiome research (55, 56), it is necessary to carefully select groups of patients that include those at risk of developing psychological disorders and those with pre-existing anxiety or depression. Hence, we recruited patients with IBD with depressive and anxious symptoms at baseline (measured by HADS score ≥ 8 on one or both two subscales), which was different from the previous studies.

Attenuating psychological problems and improving QoL is important in managing IBD because impaired QoL, depression, and anxiety are related to an increased risk of relapse and disease activity (57–59). Our study indicated that venlafaxine could improve QoL and alleviate anxious and depressive symptoms, consistent with the previous study which stated that antidepressants intervention improved QoL and mental health in patients with IBD (54, 60–62). Though the effect of venlafaxine was medium for QoL at 3 months, it became larger and statistically significant at 6 months, implying that venlafaxine therapy should be continued for at least 6 months. Of interest is that after 6-months treatment of venlafaxine, Mayo score and CDAI improved significantly as compared to the control group. These results differ from the previous longitudinal studies showing insignificant differences in CDAI between groups, probably because their participants were outpatients in remission (23, 60). Moreover, serum levels of ESR, CRP, and TNF-α decreased, and disease course improved significantly between the two groups. We are unable to distinguish whether the beneficial effect of venlafaxine on the activity of IBD is due to mood improvements or the anti-inflammatory properties of the drug. Prior studies have noted antidepressants might have direct effect on proinflammatory cytokines generated from nuclear factor-κB (NF-κB) and nitric oxide pathways, which are both involved in the pathogenesis of IBD (63). In addition, venlafaxine has been reported to play an anti-inflammatory role through downregulation of serum TNF-α, IL-1β, IL-6, and CRP in major depressive disorder (29, 30, 64) and animal researches (65–67). Another explanation of the changed IBD course may be that antidepressants play a role in altering the brain–gut interaction (68). Future trials for the understanding of the mechanism of action behind the potential demonstrated effect are warranted. However, our study did not find significant improvements on endoscopic scores, relapse rates, and frequency of biologic and corticosteroid use after treatment, which differed from that of Goodhand et al. (19) who found that antidepressants reduces relapse rates, frequency of steroids use, and number of endoscopic procedures. It may be explained by the fact that their patients have taken antidepressants for 1 year.

### Limitations

This study has several limitations. First, it was a single-center trial, and our sample size may be limited to detect the difference of endoscopic scores, relapse rate, and frequency of biologic and corticosteroid use. Hence, a multi-center approach with a large pool of patients is required. The second limitation of our study is the follow-up time of 6 months. Future studies should follow patients for at least 12 months (23). Confounders including age, gender, and current IBD treatment (like biologics) should be controlled in allocation. Third, it is also a weakness that pain and gastrointestinal symptoms were not tracked in this study as we suspect the medication would have helped with that from a brain-gut perspective.

## Conclusions

To summarize, our findings suggest that the venlafaxine may offer additional benefit to IBD with coexisting psychological problems, as evidenced by significant improvement in QoL, anxious and depressive symptoms, and the activity of IBD (measured by CDAI, Mayo scores, ESR, CRP, and TNF-α). Venlafaxine may be a promising treatment pathway for patients with IBD with anxious and depressive symptoms.

## Data Availability Statement

The original contributions presented in the study are included in the article/Supplementary Material, further inquiries can be directed to the corresponding author.

## Ethics Statement

The study was approved by West China Hospital of Sichuan University Biomedical Research Ethics Committee. The patients/participants provided written informed consent to participate in this study.

## Author Contributions

CL designed the study, contributed to data analysis and interpretation, drafted the paper, and approved its final version. PC took charge in the enrollment and randomization. YT performed the design of the study and provided critical comments on drafts. CZ contributed to data analysis and provided comments on drafts. NL, YL, and SD collected the data during the follow-up. YZ was involved in project inception, design, supervision, and manuscript revision. All authors contributed to the article and approved the submitted version.

## Funding

This work was supported by Grants from National Natural Science Fund of China (No: 81770550) and 1·3·5 project for disciplines of excellence–Clinical Research Incubation Project, West China Hospital, Sichuan University (No: 2018HXFH054).

## Conflict of Interest

The authors declare that the research was conducted in the absence of any commercial or financial relationships that could be construed as a potential conflict of interest.

## Publisher's Note

All claims expressed in this article are solely those of the authors and do not necessarily represent those of their affiliated organizations, or those of the publisher, the editors and the reviewers. Any product that may be evaluated in this article, or claim that may be made by its manufacturer, is not guaranteed or endorsed by the publisher.
