# Supplementary material for: Venlafaxine as an Adjuvant Therapy for Inflammatory Bowel Disease Patients With Anxious and Depressive Symptoms: A Randomized Controlled Trial
Source: Front Psychiatry. 2022 May 19;13:880058. doi: 10.3389/fpsyt.2022.880058 (PMC9160994; doi:10.3389/fpsyt.2022.880058)
Supplement: Supplementary file 1 [file Data_Sheet_1.docx]

| **Supplementary table S1.** Demography, disease activity and disease characteristics in IBD patients and the IBD patients separated into UC or CD, randomized to venlafaxine group or placebo group | | | | | | | | | |
| --- | --- | --- | --- | --- | --- | --- | --- | --- | --- |
| **Total Characteristics** | **IBD** | | | **UC** | | | **CD** | | |
|  | **Venlafaxine (n=25)** | **Placebo (n=20)** | ***p*** | **Venlafaxine (n=11)** | **Placebo (n=11)** | ***p*** | **Venlafaxine (n=14)** | **Placebo (n=9)** | ***p*** |
| Ages, mean (SD) year | 39.76(11.30) | 40.20(13.06) | 0.904 | 40.36 (8.87) | 42.8 (12.32) | 0.598 | 39.29 (13.21) | 37.00 (13.94) | 0.696 |
| Woman, n (%) | 11 (44.0) | 9 (45.0) | 0.947 | 5 (45.5) | 6 (54.5) | >0.999 | 6 (42.9) | 3 (33.3) | >0.999 |
| Marital status, n (%) |  |  |  |  |  |  |  |  |  |
| Married | 15 (60.0) | 13 (65.0) | 0.731 | 8 (72.7) | 7 (63.6) | >0.999 | 7 (50.0) | 6 (66.7) | 0.669 |
| Tobacco use, n (%) |  |  |  |  |  |  |  |  |  |
| Never | 11 (44.0) | 10 (50.0) | 0.92 | 4 (36.4) | 6 (54.5） | 0.570 | 7 (50.0) | 4 (44.5) | 0.705 |
| Quitted | 11 (44.0) | 8 (40.0) |  | 5 (45.5) | 5 (45.5) |  | 6 (42.9) | 3 (33.3) |  |
| Current | 3 (12.0) | 2 (10.0) |  | 2 (18.1) | 0 (0) |  | 1 (7.1) | 2 (22.2) |  |
| Mayo, mean (SD) |  |  |  | 6.64 (3.61) | 5.27 (4.00) | 0.412 |  |  |  |
| CDAI, mean (SD) |  |  |  |  |  |  | 261.43 (134.94) | 271.78 (102.51) | 0.847 |
| Disease location (%) |  |  |  |  |  |  |  |  |  |
|  |  |  |  | E1 (33.3) | E1 (18.2) | 0.738 | L1 (18.2) | L1 (11.1) | 0.615 |
|  |  |  |  | E2 (16.7） | E2 (27.3) |  | L2 (45.5) | L2 (66.7) |  |
|  |  |  |  | E3 (50.0) | E3 (54.5) |  | L3 (36.3) | L3 (22.2) |  |
| Disease behavior (%) |  |  |  |  |  |  |  |  |  |
|  |  |  |  |  |  |  | B1 (47.4) | B1 (46.0) | >0.999 |
|  |  |  |  |  |  |  | B2 (45.5) | B2 (42.9) |  |
|  |  |  |  |  |  |  | B3 (7.1) | B3 (11.1) |  |
| Extra-intestinal manifestations, n (%) |  |  |  |  |  |  | p (36.4) | p (47.1) | >0.999 |
| Surgery, n (%) | 8 (32.0) | 5 (25.0) | 0.885 | 3 (27.2) | 2 (18.2) | >0.999 | 5 (35.7) | 3 (33.3) | 0.907 |
| Medication, n (%) |  |  |  |  |  |  |  |  |  |
| Mesalazine | 8 (32.0) | 6 (30.0） | 0.839 | 7 (63.6) | 6 (54.5) | 0.526 | 1 (7.1) | 0 (0) | 0.891 |
| Corticosteroids | 1 (4.0) | 2 (10.0) |  | 0 (0) | 2 (18.2) |  | 2 (14.3) | 0 (0) |  |
| Immunomodulators | 6 (24.0) | 6 (30.0) |  | 1 (9.1) | 3 (27.2) |  | 5 (35.7) | 3 (33.3) |  |
| Biologics | 10 (40.0) | 6 (30.0） |  | 2 (18.2) | 1 (9.1) |  | 8 (57.1) | 5 (55.6) |  |
| BMI, mean (SD) | 21.54 (2.26) | 20.32 (2.63) | 0.151 | 20.76 (2.97) | 21.89 (2.17) | 0.382 | 20.08 (2.55) | 20.99 (2.45) | 0.462 |
| No differences between groups on any variables. IBD, inflammatory bowel disease; UC, ulcerative colitis; CD, Crohn’s disease; SD, standard deviation; CDAI, Crohn’s Disease Activity Index; BMI, Body Mass Index. | | | | | | | | | |

| **Supplementary Table S2.** Laboratory parameters, HADS scores and IBDQ scores at baseline | | | | | | | | | | | | | | | | | | | | | | | | |  |  |  |
| --- | --- | --- | --- | --- | --- | --- | --- | --- | --- | --- | --- | --- | --- | --- | --- | --- | --- | --- | --- | --- | --- | --- | --- | --- | --- | --- | --- |
| **Laboratory parameter** | **IBD** | | | | | |  | | **UC** | | | | | | |  | | **CD** | | | | | | | |  |  |
|  | **Venlafaxine mean (SD)** | | | **Placebo mean (SD)** | ***p*** | | |  | | **Venlafaxine**  **mean (SD)** | | | **Placebo mean (SD)** | ***p*** | | |  | | **Venlafaxine**  **mean (SD)** | | | **Placebo mean (SD)** | ***p*** | | | |  |
|  |  |  |  |  |  |  |  |  | |  |  |  |  |  |  |  |  | |  |  |  |  |  |  |  |  |  |
|  |  |  |  |  |  |  |  |  | |  |  |  |  |  |  |  |  | |  |  |  |  |  |  |  |  |  |
| WBC (×10^9/L) | | 6.38 (2.43) | 7.08 (2.37) | | | 0.395 | | | | |  | 8.52 (2.28) | 7.81 (2.21) | | 0.544 | | | | |  | 5.69 (2.11) | 7.22 (2.37) | | 0.190 | | | |
| ALB (g/L) | | 42.52 (6.97) | 44.29 (4.59) | | | 0.380 | | | | |  | 44.92 (5.52) | 44.50 (5.49) | | 0.883 | | | | |  | 41.22 (7.56) | 43.96 (3.04) | | 0.301 | | | |
| CRP (mg/L) | | 17.85 (2.74) | 16.51 (2.88) | | | 0.738 | | | | |  | 18.05 (13.60) | 14.42 (10.87) | | 0.497 | | | | |  | 17.70 (14.24) | 12.93 (9.78) | | 0.391 | | | |
| ESR (mm/h) | | 36.61 (25.08) | 26.83 (21.21) | | | 0.171 | | | | |  | 26.73 (24.01) | 21.86 (20.40) | | 0.614 | | | | |  | 44.38 (23.88) | 34.15 (30.98) | | 0.414 | | | |
| TNF-a (pg/ml) | | 55.37 (77.66) | 42.85 (50.64) | | | 0.537 | | | | |  | 55.55 (75.35) | 16.46 (21.98) | | 0.125 | | | | |  | 55.24 (82.27) | 74.38 (61.14) | | 0.530 | | | |
| IL-10 (pg/ml) | | 3.69 (1.73) | 7.05 (12.68) | | | 0.197 | | | | |  | 3.52 (1.60) | 10.31 (16.69) | | 0.209 | | | | |  | 3.93 (1.88） | 3.06 (1.11) | | 0.285 | | | |
| Haemoglobin (g/L) | | 129.88 (26.35) | 126.78 (18.89) | | | 0.091 | | | | |  | 138.50 (24.16) | 130.00 (22.83) | | 0.483 | | | | |  | 125.18 (27.39) | 121.71 (9.64) | | 0.707 | | | |
| TSH (mU/L) | | 2.25 (0.92) | 2.41 (1.25) | | | 0.815 | | | | |  | 2.05 (0.46) | 2.21 (0.83) | | 0.669 | | | | |  | 2.36 (1.10) | 2.71 (1.76) | | 0.614 | | | |
| HADS Anxiety | | 10.48 (1.96) | 9.90 (1.68) | | | 0.300 | | | | |  | 10.27 (2.15) | 9.55 (1.81) | | 0.401 | | | | |  | 10.07 (2.70) | 11.22 (2.22) | | 0.299 | | | |
| HADS Depression | | 9.24 (2.60) | 8.40 (2.09) | | | 0.248 | | | | |  | 9.18 (1.47) | 8.73 (2.37) | | 0.596 | | | | |  | 8.86 (3.66) | 7.44 (4.59) | | 0.449 | | | |
| IBDQ | | 143.04 (31.16) | 144.05 (30.97) | | | 0.914 | | | | |  | 144.45 (39.91) | 144.36 (35.13) | | 0.996 | | | | |  | 142.43 (24.87) | 143.67 (27.11) | | 0.913 | | | |
| IBD, inflammatory bowel disease; UC, ulcerative colitis; CD, Crohn’s disease; SD, standard deviation; WBC, white blood count; ALB, albumin; CRP, C-reactive protein; ESR, erythrocyte sedimentation rate; TNF-a, tumor necrosis factor α; IL-10, interleukin 10; TSH, thyroid-stimulating hormone; HADS, Hospital Anxiety and Depression Scale; IBDQ, inflammatory bowel disease questionnaire. | | | | | | | | | | | | | | | | | | | | | | | | |  |  |  |
